# Exploring the knowledge and attitudes of women of reproductive age from the general public towards egg donation and egg sharing: a UK-based study

**DOI:** 10.1093/humrep/deab157

**Published:** 2021-07-06

**Authors:** Timothy Bracewell-Milnes, James C Holland, Benjamin P Jones, Srdjan Saso, Paula Almeida, Kate Maclaran, Julian Norman-Taylor, Dimitrios Nikolaou, Nishel M Shah, Mark Johnson, Meen-Yau Thum

**Affiliations:** 1 Assisted Conception Unit, Chelsea and Westminster Hospital, London, UK; 2 Division of Surgery and Cancer, Institute of Reproductive & Developmental Biology, Imperial College London, Hammersmith Hospital Campus, London, UK; 3 The Lister Hospital, The Lister Fertility Clinic, London, UK

**Keywords:** attitudes, knowledge, disclosure, egg donation, egg sharing

## Abstract

**STUDY QUESTION:**

What are the knowledge and views of UK-based women towards egg donation (ED) and egg sharing (ES)?

**SUMMARY ANSWER:**

Lacking knowledge of the practices of ED and ES could be an influential factor in donor egg shortages, rather than negative perceptions or lack of donor anonymity and financial incentives.

**WHAT IS KNOWN ALREADY:**

The increasing age of women trying to conceive has led to donor egg shortages, with ED and ES failing to meet demand. Indeed, in recent years in the UK, ES numbers have fallen. This results in long waiting lists, forcing patients abroad for fertility treatment to take up cross border reproductive care. Previous research suggests a lack of knowledge of ED among members of the general public; however, no study has yet assessed knowledge or views of ES in the general public.

**STUDY DESIGN, SIZE, DURATION:**

Six hundred and thirty-five UK-based women over 18 years were voluntarily recruited from social media community groups by convenience sampling. The recruitment period was from February to April 2020.

**PARTICIPANTS/MATERIALS, SETTING, METHODS:**

Participants completed a previously validated questionnaire regarding female fertility, ED and ES, including knowledge, perceptions and approval of the practices and relevant legislation. This included ranking key benefits and issues regarding egg sharing. The questionnaire was completed using the online Qualtrics survey software. Statistical analysis was conducted using SPSS.

**MAIN RESULTS AND THE ROLE OF CHANCE:**

Regarding knowledge of ED and ES, 56.3% and 79.8%, respectively had little or no prior knowledge. Upon explanation, most approved of ED (85.8%) and ES (70.4%). A greater proportion of respondents would donate to a family member/friend (49.75%) than to an anonymous recipient (35.80%). Overall, ES was viewed less favourably than ED, with ethical and practical concerns highlighted. Women aged 18–30 years were significantly more likely to approve of egg donation practice compared to those aged >30 years (*P* < 0.0001). Those against ES found fears of financial coercion or negative psychological wellbeing the most concerning. About 35.8% and 49.7% would personally consider anonymous and known ED, respectively, whilst 56.7% would consider ES. Those answering in favour of egg sharing were significantly more likely to give higher benefit ratings compared to those against the practice (*P* < 0.001). Most agreed (55.8%) with and were not deterred to donate (60.1%) by the ‘Disclosure of Donor Identity’ legislation. Only 31.6% agreed with the compensatory cap; however, 52.7% would not be more motivated to donate by an increased cap.

**LIMITATIONS, REASONS FOR CAUTION:**

There were several limitations of the study, including the use of convenience sampling and the voluntary nature of participation opening the study up to sampling and participation bias. Finally, closed questions were predominantly used to allow the generation of quantitative data and statistical analysis. However, this approach prevented opinion justification and qualitative analysis, limiting the depth of conclusions drawn.

**WIDER IMPLICATIONS OF THE FINDINGS:**

To our knowledge, this is the first study to survey the general public’s knowledge and views of ED/ES using a previously validated questionnaire. The conclusion that lack of knowledge could be contributing to the current donor shortfall in the UK demonstrates that campaigns to inform women of the practices are necessary to alleviate donor oocyte shortages.

**STUDY FUNDING/COMPETING INTEREST(S):**

No external funds were used for this study. The authors have no conflicts of interest.

**TRIAL REGISTRATION NUMBER:**

NA.

## Introduction

Infertility affects one in seven couples in the UK, resulting in 2% of all live births being conceived using ART ((HFEA), 2014; HFEA, 2019). The advent of IVF allowed for the development of egg donation, a technique first successfully performed in 1984 (Lutjen *et al*., 1984). This process provides donor eggs to women who are unable to conceive with their own eggs, most commonly older women and those with primary ovarian insufficiency (POI) (Bracewell-Milnes *et al*., 2016). Donor egg usage has been steadily increasing since its introduction with 5% of IVF cycles in the UK currently using donor eggs, and 18% of cycles in women over 40 years using donor eggs and partner sperm (DEPS) (HFEA, 2019). This is unsurprising as UK data show women aged 40–42 years had a live birth rate (LBR) four times higher using donor eggs compared to autologous (35% vs 9%); and women aged 43–44 years had an almost 10-fold increase in LBR using donor eggs instead of their own (32% vs 4%) (HFEA, 2018). In contrast to alternative family building options, DEPS cycles give the recipient the opportunity to experience being pregnant and a genetic link between the offspring and their partner (Applegarth *et al*., 1995).

Different countries have contrasting practices regarding gamete donation, with countries, such as the USA permitting commercial payments to the donor for her eggs (Partrick *et al*., 2001). In the UK, commercial payments are illegal, with patients who donate eggs only entitled to a ‘compensatory’ payment of £750, to cover resulting travel expenses and time taken off work ((HFEA), 2014). There are two distinct groups of egg donors: (i) ‘non-patient donors’, who can be known donors (the egg donor is known to the recipient), altruistic donors (anonymous donation without financial reward) or commercial donors (donation for financial reward); and (ii) ‘patient or egg share donors’, where fertility patients give a proportion of their eggs in exchange for subsidized fertility care. Other than commercial donors, all other donation types are available in the UK.

Demand for egg donation has been on the rise globally, with an increase of 49% of DEPS cycles in the UK since 2011 (HFEA, 2018). Unlike sperm donation, the process of egg donation involves multiple clinic visits, daily injections of high-dose ovarian stimulation and invasive procedures, such as transvaginal egg retrieval. In addition to this, UK legislative changes in 2005 require that any gamete donor consents to their identity being released to any resulting offspring when they turn 18 years of age ((HFEA), 2005). It is therefore unsurprising that relatively few women are prepared to donate their eggs on a purely altruistic basis, and therefore demand for donor eggs exceeds supply in the majority of UK fertility clinics. This has led to couples seeking fertility treatment abroad, a process known as ‘cross-border reproductive care’ (CBRC), where donor eggs and choice may be more readily available and regulations less rigorous (Culley *et al*., 2011).

Women can attempt to avoid such long waiting lists by sourcing their own donor eggs through family and friends. Another solution recipient women have in the UK is to pursue eggs donated by ‘egg sharers’, a process regulated in the UK since 1998, and now being performed in many western countries, such as Australia and the USA (Blyth, 2002, Gurtin *et al*., 2012). Egg sharing has the potential to combat the international shortage of donor eggs; however, in the UK, the number of egg sharers has fallen, with a 50.1% decrease between 2011 and 2016 (HFEA, 2019).

The egg sharing scheme was criticized by some experts when it was introduced and the practice has been debated over the years (Blyth, 2001, 2002). There are definite benefits; first, it allows patients to access IVF who do not have the option of government funded treatment and cannot afford to self-fund. Second, the egg share donor requires IVF treatment for her own fertility needs, so no third party is undergoing this invasive treatment unnecessarily. Nevertheless, concerns regarding egg sharing have been put forward. First, it has been suggested the egg share donor could be negatively impacting her chance of success by giving away half her eggs (Bracewell-Milnes *et al*., 2019). Second, there is concern for the negative psychological impact on the donor if her own treatment fails, with this issue potentially worsened if the recipient conceived their genetic children (Johnson, 1999). Third, theoretical issues have been raised regarding the donor’s quality of consent, in that she is only agreeing to donate so she can access much desired IVF (Lieberman *et al*., 2008).

Studies investigating the general public’s knowledge of egg donation have reported relatively poor awareness of the practice (Baykal *et al*., 2008; Stevens and Hayes, 2010; Straehl *et al*., 2017). Interestingly, even amongst infertile populations undergoing IVF, perceived knowledge has been shown to be poor, with patients reporting ‘very little knowledge’ about egg donation (Stevens and Hayes, 2010; Straehl *et al*., 2017). Nevertheless, limited or variable knowledge about egg donation did not appear to obstruct an individual’s hypothetical intention to donate eggs or their ability to express opinions on the topic (Chliaoutakis *et al*., 2002; Culley *et al*., 2007). Studies in western populations have shown generally positive attitudes towards egg donation (Isikoglu *et al*., 2006; Lee *et al*., 2017). However, this was contradicted by studies of different ethnicities and religious backgrounds, including Asian populations and Muslim religious background, where the majority of participants reported the use of donor gametes to be ‘socially unacceptable’ (Bharadwaj, 2003; Culley *et al*., 2007; Purewal and van den Akker, 2010; Stevens and Hayes, 2010). A UK study also specifically reported that British Caucasian women were more likely to agree to egg donation compared to British Asian women (Purewal and van den Akker, 2006).

Whilst the knowledge and attitudes towards egg donation and egg sharing amongst donors, recipients and healthcare professionals have been studied, there has been no study of the UK general public. This is especially relevant, as these women represent potential egg donors who could bridge the gap between supply and demand in the UK. This study aims to investigate the UK general public’s knowledge and perceptions of female fertility, as well as their knowledge and attitudes towards egg donation and egg sharing. Secondary aims were to explore public opinion of the 2005 legislative change towards donor anonymity. This knowledge could identify potential methods for increasing the number of women coming forward for egg donation. In addition, population delay in childbearing and the increasing use of IVF means women from the general population may become egg sharers or recipients in the future, making the findings from this study of significant interest.

## Material and methods

### Study design

An in-depth survey investigating the general public’s knowledge and attitudes towards egg donation and egg sharing was designed. The format of the questionnaire (see Supplementary Material) was based on the previously validated and published studies investigating the attitudes towards face and uterine transplantation, as well as a survey on healthcare professionals views on egg donation, published by the same group (Clarke *et al*., 2007; Saso *et al*., 2015; Bracewell-Milnes *et al*., 2019). The survey was modified to assess the knowledge and attitudes of fertility, egg donation and egg sharing among female members of the general public. The questions participants were asked to consider were selected based on their identification in previous published systematic reviews that the authors performed (Bracewell-Milnes *et al*., 2016, 2017; Platts *et al*., 2019). The questionnaire was piloted on fertility consultants, nurses and counsellors within the egg donation team at a London based fertility clinic, alongside 10 fertility patients who were undergoing fertility treatment. Feedback was noted and minor revisions to the questionnaire were made, namely medical jargon and questions deemed unnecessary to aid survey completion.

### Study participants

The inclusion criteria for the questionnaire were to be female, over 18 years of age and living in the UK. This initial check was the first question of the survey, with only those fulfilling the above criteria able to continue and answer the survey questions. The survey consists of 46 simple close-ended questions that took 15 min to complete. The questionnaires were distributed online using the Qualtrics survey tool. A link to the survey and a brief summary of the purpose of the study were posted on social media neighbourhood community groups based throughout the UK. These were general community groups with no link to fertility groups or networks. The posts were made on the condition of the group administrator’s approval. Of the 105 posts made, 27 were approved by the administrator. Responses were received on a purely voluntary basis, with no incentives offered to participants. The questionnaire contained five main sections: (i) demographic and personal information, (ii) knowledge of female fertility, (iii) egg donation, (iv) egg sharing and (v) UK legislation.

### Data collection and analysis

Data collection occurred over a 45-day period from 15 March to 29 April 2020. Data were collated and exported using the Qualtrics survey tool, with statistical analysis of the generated quantitative data performed using SPSS. Comparisons between categorical data were analysed using Pearson’s *χ*^2^ test. Distributions of mean grades from the ranking of benefits and issues of egg sharing were assessed using the Mann–Whitney *U* testing. Statistical significance was set as *P* < 0.05.

### Ethical approval

Ethical approval for this study was provided by ‘London Riverside Research Ethics Committee’, Research Ethics Committee (REC) reference: 17/LO/1491.

## Results

Respondent characteristics are summarized in Table I. A total of 635 participants took part in the study. Five hundred and twenty-three participants completed all the questions meaning 134 responses left one or more question unanswered, these responses were not excluded from analysis. All respondents were female, with the majority aged 18–25 years (35.8%), although there was a relatively even spread among the different age groups (Table I). The vast majority of participants were Caucasian (91.0%), and the majority stated they were ‘not religious’ (52.1%). Regarding relationship status, the respondents were evenly spread, with 26.5% single, 35.3% ‘in a relationship’ and 38.2% married. About 91.8% of respondents described their sexual orientation as heterosexual. The majority of participants reported their educational level to be a university degree (42.6%), worked full-time (49.5%) and earned an annual salary of < £30 000 (70.3%).

**Table I:** Sample population demographics.

Characteristics (total no. of respondents, n)	Respondents	
	%	n
Age, years (657)		
18–25	35.8	235
26–30	12.8	84
31–35	10.1	66
36–40	13.2	87
41–45	10.7	70
>45	17.5	2
Ethnicity (657)		
White British	83.7	550
With other	7.3	48
Mixed ethnicity White/Black	1.8	12
Mixed ethnicity White/Asian	1.5	10
Mixed ethnicity Other	0.6	4
Asian Indian/Pakistani/Bangladeshi	1.9	13
Asian Chinese	0.4	3
Asian Other	0.3	2
Black African/Caribbean	1.3	9
Arabic	0.2	1
Other Ethnicity	0.8	5
Relationship status (657)		
Single	26.5	174
In a relationship unmarried	35.3	232
Married	38.2	251
Sexual orientation (657)		
Heterosexual	91.5	601
Homosexual	2.3	15
Bisexual	5.8	38
Other	0.5	3
Existing children (657)		
Yes	48.7	320
No	51.3	337
Religious background (657)		
None	52.1	342
Christian	43.8	288
Muslim	1.2	8
Jewish	0.6	4
Hindu	0.9	6
Other	1.4	9
Education level (657)		
GCSEs	12.6	83
A-levels	14.2	93
College diploma/apprenticeship	14.2	93
University degree	42.6	280
Postgraduate degree	16.4	108
Employment status (657)		
Full time	49.5	325
Part time	18.7	123
Student	23.9	157
Housewife/housework	5.3	35
Unemployed	2.6	17
Annual salary (657)		
<£30 000	70.3	462
£30–50 000	23.6	155
£50–100 000	5.2	34
>£100 000	0.9	6

### Knowledge and attitudes towards egg donation

Prior to answering the survey, the majority of respondents had ‘little’ or ‘no knowledge’ surrounding egg donation (56.4%), with only 8.8% stating ‘significant knowledge’ on the topic (Table II). Once an impartial informative statement about the egg donation programme was provided, 86.0% responded that they agreed with egg donation, with only 3.2% stating they disagreed with this process (Table II). Prior knowledge of egg donation had no effect on whether participants agreed with it (*χ*^2^ testing, *P* = 0.660). However, those aged 18–30 years were significantly more likely to approve of egg donation practice compared to those aged >30 years (*P* < 0.05).

**Table II:** Summary of respondents’ answers about egg donation.

Characteristics (total no. of respondents, n = 591)	Percent	n
Knowledge about egg donation prior to answering the questionnaire		
No knowledge	15.4	91
Little knowledge	41.0	242
Some knowledge	34.9	206
Significant knowledge	8.8	52
Do you agree with the principle of egg donation?		
Yes	86.0	508
No	3.2	19
Unsure	10.8	64
Would you consider donating your eggs altruistically as an anonymous donor?		
Yes	35.9	212
No	36.7	217
Unsure	27.4	162
Would you consider donating your eggs to a close friend or relative as a known donor?		
Yes	49.8	294
No	27.2	161
Unsure	23.0	136
If you were to donate your eggs hypothetically, what would be your main motivation?		
Altruism	29.0	171
Financial	7.7	46
Family/friend having fertility problems	58.8	348
Passing on my genetic material	1.4	8
Developing a relationship with an infertile couple	3.2	18
If you were to donate your eggs hypothetically, what would be your main concern?		
Medical procedures	43.3	213
Potential future contact with child	34.2	168
Recipient may be too old	0.6	3
Recipient may be in a same-sex relationship	1.0	5
The donation not working	16.5	81
Taking time off work	3.5	17
Religious reasons	1.0	5
If you were unable to have a child and your only realistic option to conceive was through an egg donor, would you pursue egg donation?		
Yes	56.0	331
No	17.8	105
Unsure	26.2	155

While most agreed with the principle of egg donation, only 35.8% said they would consider donating their eggs altruistically as an anonymous donor. However, 49.8% would consider donating their eggs to a family member or friend. When questioned on their main motivating factor in the hypothetical case of donating their eggs, more than half answered ‘family/friend having fertility problems’ (58.8%), with altruism (7.7%) and financial reasons (7.7%) being the other most popular motivating factors (Table II). The main hypothetical concerns raised were the medical procedures endured (43.4%), potential future contact with the child (34.3%) and the donation not working (16.5%) (Table III). When asked if they would only be able to get pregnant by using donor eggs, 56.0% said they would pursue this option, with 17.8% answering ‘no’ (Table II).

**Table III:** Summary of respondents’ answers about egg sharing.

Characteristics (total no. of respondents, n)	Percent	n
Knowledge about egg sharing prior to answering the questionnaire (571)		
No knowledge	54.6	312
Little knowledge	25.0	143
Some knowledge	14.2	81
Significant knowledge	6.1	35
Do you agree with the principle of egg sharing? (571)		
Yes	73.4	419
No	9.1	52
Unsure	17.5	100
Do you believe egg sharing could be a viable solution to the worldwide shortage of donor eggs? (571)		
Yes	60.6	346
No	12.6	71
Unsure	26.8	153
Do you see an ethical difference between egg sharers receiving free fertility treatment and a commercial donor being paid to donate? (523)		
Yes	56.8	297
No	22.0	115
Unsure	21.2	111
Hypothetically, if you needed IVF to have a child would you consider egg sharing? (523)		
Yes	56.8	297
No	21.9	115
Unsure	21.2	111

### Knowledge and attitudes towards egg sharing

Unsurprisingly, the majority of respondents had ‘little’ or ‘no knowledge’ surrounding the egg sharing programme (80.7%) (Table III). After a brief impartial outlining of the procedure, participants were asked if they agreed with the principle of egg sharing, with 70.4% agreeing, 9.1% disagreeing and 17.5% unsure (Table III). About 60.6% of participants felt egg sharing could be a viable solution to the worldwide shortage of donor eggs (Table III). About 56.8% saw an ethical difference between egg sharers receiving free fertility treatment and a commercial donor being paid to donate, with 22.0% seeing no difference (Table III). Overall, those in favour of egg donation were significantly more likely to approve the practice of egg sharing (*P* < 0.001).

**Figure 1.**
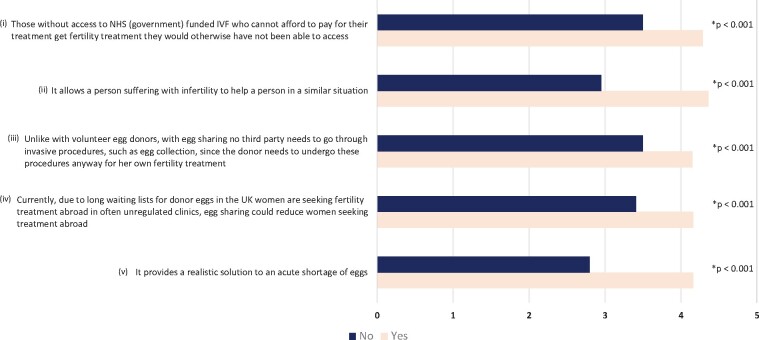
**The distributions of significance ratings for each benefit of egg sharing.** Bars show mean responses on a scale ‘1’ = ‘Not significant’ to ‘5’ = ‘Very significant’, stratified by answers to the question ‘In your opinion, should egg sharing take place?’ (‘Yes’ [n = 336] or ‘No’ [n = 53]). Statistically significant differences in ‘significance rating’ distributions between those answering ‘Yes’ or ‘No’ upon Mann–Whitney *U* testing are shown, with ‘*’ representing a *P*-value <0.001.

Respondents were asked to grade the importance of five potential benefits of egg sharing. Each benefit was given a rank between 1 (least significant) and 5 (most significant), and the mean scores given to the various benefits are summarized in Table IV. For all five benefits, the modal rating was ‘5’. All mean scores were >4 other than benefit (v) ‘egg sharing provides a realistic solution to an acute shortage of eggs’, with this benefit’s mean score 3.95. Allowing access to IVF to those who did not qualify for government funding and would not otherwise be able to afford IVF was found to be the most significant benefit. Mean rankings of egg sharing benefits were stratified by the response to the question as to whether egg sharing should take place. Statistical analysis revealed that those in favour of egg sharing were significantly more likely to give higher benefit ratings compared to those against the practice (Fig. 1, Mann–Whitney *U* test, *P* < 0.001).

**Table IV:** Mean scores given to benefits surrounding egg sharing according to their significance.

Benefits	Mean score ± SD
Those without access to NHS (government) funded IVF who cannot afford to pay for their treatment get fertility treatment they would otherwise have not been able to access	4.15 ± 1.04
It allows a person suffering from infertility to help a person in a similar situation	4.15 ± 1.05
Unlike with volunteer egg donors, with egg sharing no third party needs to go through invasive procedures, such as egg collection, since the donor needs to undergo these procedures anyway for her own fertility treatment	4.01 ± 1.03
Currently, due to long waiting lists for donor eggs in the UK women are seeking fertility treatment abroad in often unregulated clinics, egg sharing could reduce women seeking treatment abroad	4.00 ± 1.11
It provides a realistic solution to an acute shortage of eggs	3.95 ± 1.09

1 = least significance, 5 = most significance. Mean and standard deviation (SD) calculated using only responses where all benefits were scored (n = 523).

**Figure 2.**
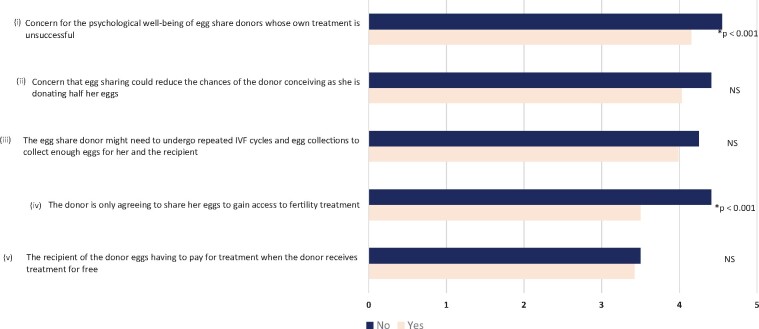
**The distributions of significance ratings for each concern with egg sharing.** Bars show mean responses on a scale where ‘1’ = ‘Not significant’ and ‘5’ = ‘Very significant’, stratified by answers to the question ‘In your opinion, should egg sharing take place?’ (‘Yes’ [n = 336] or ‘No’ [n = 53]) Statistically significant differences in ‘significance rating’ distributions between those answering ‘Yes’ or ‘No’ upon Mann–Whitney U testing are shown, with ‘*’ representing a *P*-value <0.001 and ‘NS’ representing no statistical significance.

Participants were also asked to grade the importance of five issues surrounding egg sharing. Each issue was given a grade between 1 (least significant) and 5 (most significant), with the results summarized in Table V. ‘Concern for the psychological well-being of egg share donors whose own treatment is unsuccessful’ and ‘concern that egg sharing could reduce the chances of the donor conceiving as she is donating half her eggs’ were the only two responses to rank with a mean score >4. Mean rankings of egg sharing issues were stratified by the response to the question as to whether egg sharing should take place. Statistical analysis revealed that those against egg sharing were significantly more likely to give higher issue ratings to those in favour of the practice for question (i) and (iii) (Fig. 2, Mann–Whitney *U* test, *P* < 0.001).

**Table V:** Mean scores given to issues surrounding egg sharing according to their significance.

Issues	Mean score ± SD
Concern for the psychological well-being of egg share donors whose own treatment is unsuccessful	4.14 ± 0.99
Concern that egg sharing could reduce the chances of the donor conceiving as she is donating half her eggs	4.14 ± 0.99
The egg share donor might need to undergo repeated IVF cycles and egg collections to collect enough eggs for her and the recipient	3.95 ± 0.97
The donor is only agreeing to share her eggs to gain access to fertility treatment	3.57 ± 1.27
The recipient of the donor eggs having to pay for treatment when the donor receives treatment for free	3.57 ± 1.27

1 = least significance, 5 = most significance. Mean and standard deviation (SD) calculated using only responses where all issues were scored (n = 523).

**Figure 3.**
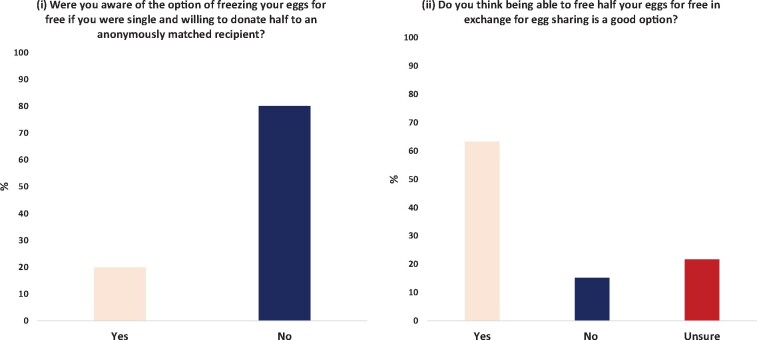
**The general public’s views on potentially using the egg sharing programme to access fertility preservation (n = 523)**.

Regarding the use of egg sharing to access fertility preservation, participants were asked whether they were aware they could participate in egg sharing and then freeze a proportion of their eggs if they were single or not ready to start a family, with 80.1% of respondents unaware of this option (Fig. 3). When asked if they thought being able to potentially preserve their fertility for free in exchange for sharing their eggs was a good option, 63.3% of the those surveyed answered ‘yes’, 15.1% answered ‘no’ and 21.7% were ‘unsure’ (Fig. 3).

### Attitudes towards UK legislation and compensatory caps

**Figure 4.**
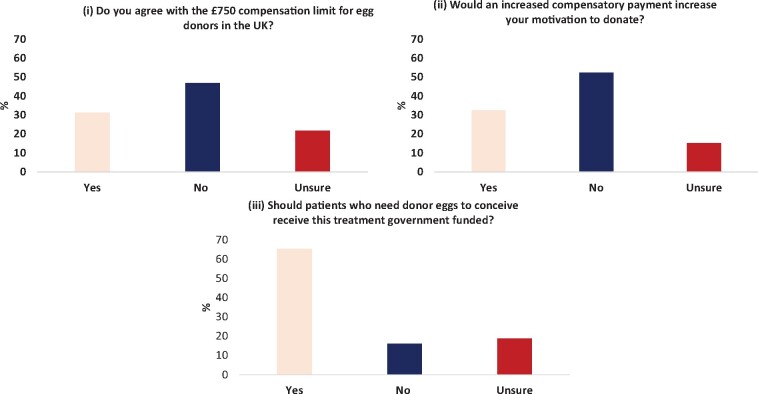
**Attitudes of the general public towards compensatory payments and government funding of egg donation cycles**.

Most answered ‘no’ (46.9%) when asked if they felt the £750 compensatory limit for donating eggs was sufficient, with 31.3% answering ‘yes’ and 21.8% ‘unsure’ (Fig. 4). However, a significant majority of patients would not be more motivated to donate if this compensatory limit was increased (52.6%), with only 32.5% answering ‘yes’ to this question (Fig. 4). Approval or opposition to this compensatory limit was not associated with annual salary (*P* = 0.41). Those under 30 years of age were significantly less likely to approve of the compensatory cap (*P* < 0.001) and were significantly more likely to be more motivated to donate if this cap were increased (*P* < 0.001), when compared with those aged over 30 years.

A significant majority (65.3%) of those surveyed felt that those requiring donor eggs to conceive should have their treatment funded by the government, with only 16.1% opposed this (Fig. 4). Those in opposition to egg donation were significantly less likely to approve government funding of egg donor IVF treatment, compared to those in favour of egg donation (*P* < 0.001).

When given an explanation and asked about the ‘Disclosure of donor information’ legislation, the majority of women agreed (55.8%), with 19.1% disagreeing and 25.0% unsure (Table VI). The majority (59.2%) also stated that this legislation would not stop them from donating their eggs. Those in opposition to the 2005 legislation were statistically more likely to not donate as a result (*P* < 0.001). A slim majority of participants answered ‘no’ to future contact with the couple they donated to (31.6%) or the child resulting from their donation (31.6%), compared to those answering ‘yes’ (Table VI). However, the majority were unsure regarding future contact with the couple (43.3%) and resulting offspring (45.4%) (Table VI).

**Table VI:** Summary of respondents’ attitudes towards disclosure and potential future contact with donor offspring.

Characteristics (total no. of respondents = 591)	Percent	n
Do you agree with the 2005 ‘Disclosure of Donor Information’ legislation?		
Yes	55.8	330
No	19.1	113
Unsure	25.0	148
Would the 2005 ‘Disclosure of Donor Information’ legislation stop you from donating eggs?		
Yes	20.6	122
No	59.2	350
Unsure	20.1	119
Hypothetically if you donated your eggs, would you want future contact with the couple you donated to?		
Yes	25.0	148
No	31.6	187
Unsure	43.3	256
Hypothetically if you donated your eggs, would you want future contact from any children resulting from the donation?		
Yes	23.0	136
No	31.6	187
Unsure	45.4	268

## Discussion

To our knowledge, this is the first study that has investigated knowledge and attitudes of the practice of egg sharing amongst the general public. This study also aimed to understand the general public’s knowledge and attitudes towards egg donation, and the issues of anonymity.

### Egg donation

Knowledge regarding egg donation was generally poor, with 56.4% of respondents indicating ‘little or no knowledge’ surrounding this practice, which is consistent with the literature (Urdapilleta *et al*., 2001; Chliaoutakis *et al*., 2002; Isikoglu *et al*., 2006; Baykal *et al*., 2008; Straehl *et al*., 2017). A study of the Turkish general public reported less than one-third had any previous knowledge pertaining to egg donation and its potential role in assisted reproduction (Isikoglu *et al*., 2006). However, after a brief description, 86.0% agreed with its practice. Additionally, 35.9% said they would consider anonymous egg donation themselves, with 49.75% stating they would consider donating to a close friend or family member. This study is interesting as it contradicts the other studies, where the majority of women from Asian and Muslim backgrounds found egg donation ‘socially unacceptable’ (Bharadwaj, 2003; Culley *et al*., 2007; Purewal and van den Akker, 2010; Stevens and Hayes, 2010).

Regarding motivations to donate, 87.8% of our cohort was motivated by ‘altruism or ‘family/friend having fertility problems’, and only 7.7% cited financial motives. This is perhaps unsurprising in a country where monetary payments for donations are illegal and payments are capped as compensatory. However, respondents were not informed of this at this stage of the questionnaire, implying there may be a cultural apprehensiveness towards payment for gamete donation in the UK. Other studies consistently report altruism to be the predominant motivation among the general public or potential donors (Purewal and van den Akker, 2010; Stevens and Hayes, 2010; Svanberg *et al*., 2012; Gezinski *et al*., 2016; Bakker *et al*., 2017). A systematic review by Platts *et al*. (2019) reported that 82–98% of prospective egg donors described altruism as the main motivating factor that would result in them donating.

The majority of studies in the literature showed generally negative attitudes towards payment of egg donors (Oskarsson *et al*., 1991; Kazem *et al*., 1995; Lyall *et al*., 1995; Westlander *et al*., 1998; Waldby *et al*., 2013; Pennings *et al*., 2014; Waldby, 2015). It could be argued that it is difficult to ascertain whether financial motivations could be a motivation in countries where financial payment is prohibited or capped, such as in the UK. Indeed, Pennings *et al.* (2014) studied the motivations to potentially donate eggs in 11 different European countries and reported financial payments to be significantly less of a motivating factor in countries where it is not an option, compared to those in which it is (Pennings *et al*., 2014). Other studies have reported financial payments to play a significant motivating influence among potential donors, with this differing depending on the country of study, namely the USA (Lindheim *et al*., 2001; Stevens and Hayes, 2010; Gezinski *et al*., 2016; Lee *et al*., 2017). One study reported 90% of respondents supported paying for donor eggs, with an appropriate compensation no more than $10 000 (Lee *et al*., 2017). Lindheim *et al.* (2001) investigated the motivations of potential egg donors of similar demographics and compared groups who could receive $2500 or $5000 to donate (Lindheim *et al*., 2001). The financial incentives were significantly higher in those receiving $5000 (68%) than $2500 (39%) (Lindheim *et al*., 2001). In countries where payments are prohibited, there are significantly fewer donors, implying financial motivation is a significant motivating factor (Platts *et al*., 2019).

The main concern in our study regarding egg donation was the medical procedures involved (43.3%). This is consistent with other studies of the general public which reported 33–67% of participants had concerns regarding the invasiveness of the medical procedures and risks involved (Kan *et al*., 1998; Svanberg *et al*., 2003; Stevens and Hayes, 2010; Gezinski *et al*., 2016). Interestingly, one study reported potential donors were more likely to donate if they could speak to women who had already donated eggs (Svanberg *et al*., 2003). Only 0.6% of our participants reported their main concern to be the recipient ‘being too old’ and 1.0% reported the recipient being in a same-sex relationship. This is consistent with a study where 100% of respondents were in favour of donation to female same-sex couples, and 97% to male same-sex couples (Zweifel *et al*., 2006).

### Egg share donors

Knowledge of egg sharing in the UK is lacking, with 80.7% of respondents having little or no knowledge of the practice. This is unsurprising considering 63.1% of healthcare professionals in another study reported little or no knowledge of egg sharing (Bracewell-Milnes *et al*., 2019). This translated to only 16.5% of those surveyed who were able to refer a patient for egg sharing having done so (Bracewell-Milnes *et al*., 2019). When briefed on egg sharing, 73.4% of the general public agreed with it, with almost two-thirds of respondents feeling egg sharing could represent a viable solution to the worldwide shortage of donor eggs. This is a similar level of support towards egg sharing among the general public in the UK, as shown by healthcare professionals, in whom 78.2% reported agreement (Bracewell-Milnes *et al*., 2019). The combination of the general public and healthcare professionals having poor knowledge of the practice is likely translating into poor referral rates for egg sharing and patient self-referrals, which could be one of the contributing factors into the current shortfall in donors, especially given the high approval ratings after information was given.

Interestingly, respondents in our study reported all of the benefits highly; with ‘allowing those access to fertility treatment who otherwise would not be able to access it’, and ‘the lack of a third party undergoing invasive procedures to donate’, the highest ranking benefits, which is consistent with previous research (Bracewell-Milnes *et al*., 2016, 2017, 2019; Purewal and van den Akker, 2009). The concerns rated most significantly amongst our cohort were ‘fears of egg sharing reducing the chance of success for the egg sharer’, and ‘concern for the psychological well-being for the egg sharer if her own treatment was unsuccessful’, with the knowledge her anonymously matched recipient may have conceived. These concerns have been raised consistently in theory as expert opinion (Blyth, 2001, 2002; Johnson, 1999), and also in a survey of healthcare professionals (Bracewell-Milnes *et al*., 2019). This shows the respondents have a good understanding of the concept of egg sharing, as they cited similar positive and negative aspects surrounding the programme as medical professionals and experts in the field. Perhaps unsurprisingly in this study, those in favour of egg sharing rated these concerns as less significant than those against egg sharing.

Regarding the impact egg sharing could have on success rates, the majority of studies have reported no difference in live birth rate between egg share donors and age-matched standard IVF patients (Thum *et al*., 2003; Oyesanya *et al*., 2009; Check *et al*., 2012). Numerous studies have investigated the psychological well-being of egg share donors, and consistently reported positive attitudes towards treatment experience, and low levels of regret, even when their own treatment was unsuccessful (Bracewell-Milnes *et al*., 2016, 2017).

Regardless of the concerns raised by our cohort, only 21.9% answered ‘no’ when asked if they would consider egg sharing if they may hypothetically require it in the future, implying the positive aspects of this practice outweigh the concerns raised. This is true also regarding the potential use of egg sharing for fertility preservation. Only 19.9% of respondents were aware that by participating in egg sharing they could undergo social egg freezing for minimal cost, and only 15.1% of our cohort thought this was not a good option to be available. Indeed, other studies have reported financial cost as the most significant barrier to oocyte cryopreservation for non-medical reasons (Daniluk and Koert, 2016; Santo *et al*., 2017; Hurley *et al*., 2018). Other studies reported that up to 71–73% of women would likely go ahead with social egg freezing if the cost were subsidized by the government or employer (Tan *et al*., 2014; Ikhena-Abel *et al*., 2017; Mahesan *et al*., 2019). With financial burdens one of the most significant barriers to women accessing this method of fertility conservation, egg sharing is another option to government or employer spending. With the age of women achieving first-time motherhood increasing in the UK over recent decades (ONS, 2020), allowing more women to access social egg freezing would reduce the number of women suffering involuntarily childlessness, or those requiring a donor egg to conceive in the future.

### Legislation surrounding egg donation

With the passing of the ‘Disclosure of Donor Identity’ legislation in 2005 in the UK, it was feared there would be a significant reduction in anonymous oocyte donor recruitment. The majority of participants agreed with this legislature, with only 19.1% stating open opposition and only 20.6% stating this legislature would stop them from potentially donating. This data are supported by two systematic reviews on the attitudes towards donor anonymity, with the majority supporting donor identifying data (Bracewell-Milnes *et al*., 2016; Platts *et al*., 2019). Indeed, HFEA figures saw an initial decline when this legislation was introduced, but have seen a consistent rise in numbers of altruistic oocyte donation in recent years ((HFEA), 2014). Despite this, the slight majority of respondents would not want contact with the couple or child they donated to in a hypothetical scenario, which is again consistent with other studies (Platts *et al*., 2019). This implies that although the general public and potential donors agree with the principal of non-anonymous egg donation, the concept of meeting resulting offspring remains overwhelming. In 2023, the first offspring conceived from donor gametes will be able to contact their donor. Research looking at the longitudinal psychological outcomes of egg donors following donation will be pivotal in defining future recruitment of donor eggs.

Regarding compensatory payments, the majority of our cohort were undecided or against the cap of £750, with views on the cap independent of the annual salary of the respondent. Despite this, most respondents stated an increase in this compensatory cap would not increase their motivation to donate their eggs, and very few respondents answered that financial gain would be their main motivation to donate. These findings suggest that increasing the compensatory cap or allowing commercial egg donation in the UK may not significantly increase the uptake of donors. Other studies in countries in which commercial payment for donor eggs is not permitted found similar attitudes towards monetary payments (Oskarsson *et al*., 1991; Kazem *et al*., 1995; Lyall *et al*., 1995; Waldby *et al*., 2013). The impact on recruitment rates is not the only consideration countries should have when considering the ethics of financial payments. However, the fact it seems unlikely to result in an increase in recruitment rates is an interesting finding.

The majority of the cohort was in favour of patients who require donor eggs to conceive having their treatment government funded. In the UK, fertility treatment funding varies by clinical commissioning group (CCG); however, the majority would not fund treatment involving a donor egg, especially in women over 40 years.

### Potential clinical utilization of this survey

The average age of women having their first child in the UK has risen significantly since the 1970s; in 1975 the mean female age of parenthood was 26.4 years, compared to 30.7 years in 2019 (ONS, 2020), leading to an increased demand for donor eggs. Despite this need, egg donation numbers fall short of demand in the UK currently, and indeed egg share numbers have dropped in recent years, with experts in the field unclear for the reasons behind this (HFEA, 2018).

The reasons for low egg donation numbers and falling egg share numbers in the UK are complicated, multi-factorial and difficult to explain, requiring further in-depth research. Since healthcare professionals, participating patient donors and the general public all felt overwhelmingly positive about the egg sharing scheme, there must be other explanations for the recent fall in numbers in the UK. Therefore, one possible contributory factor for the low number of egg sharers in the UK is a lack of knowledge about the programme among the general public and healthcare professionals, highlighted by our data. Indeed, only 16.5% of healthcare professionals who could refer a patient for egg sharing had done so (general practitioners, obstetricians and gynaecologists and fertility specialists), with the vast majority citing lack of knowledge for the reason they had not referred (Bracewell-Milnes *et al*., 2019).

An instrumental tool to increase egg share numbers could be to educate healthcare professionals and the general public about the existence of egg sharing and the research relating to it. This would not only increase potential donor awareness of the egg sharing scheme but give healthcare professionals the tools to address concerns that may prevent women from participating. It should also lead to an increased referral rate for egg sharing, resulting in an increasing number of women accessing fertility specialists with sufficient expertise to answer all their questions in sufficient detail. It is particularly important to identify and contest misconceptions regarding female fertility, egg donation and egg sharing within the general public, as these misunderstandings may lead to a deterrence to donate.

There is precedent to suggest that increasing awareness of egg sharing would improve uptake, since the HFEA attributes the increase in voluntary egg donor registration to a heightened awareness as a result of a marketing drive (HFEA, 2011). Indeed, a 2005 TV and radio advertising campaign by the National Gamete Donation trust (NGDT) increased enquiries from potential donors by over 500% across the following 6 months (BioNews, 2005). Implementing a similar strategy may have a comparable impact on egg sharing numbers, since currently <20% of egg share donors find out about the scheme by advertising (Bracewell-Milnes *et al*., 2017). The significantly increasing popularity of social media over the last decade may allow for greater viewership of online advertising, thus potentially replicating or surpassing the success of these previous campaigns.

### Limitations of study and recommendations for future research

This is the first study to survey the general public’s knowledge and views of egg sharing. Over 600 women participated in the study, producing statistically significant data. In addition, the study’s survey was modified from a validated questionnaire which was used to assess attitudes in three studies published previously in peer-review journals (Clarke *et al*., 2007; Saso *et al*., 2015; Bracewell-Milnes *et al*., 2019), and has allowed for the development of insights into numerous factors potentially influencing egg donation and egg sharing rates in the UK.

However, there were several limitations of the study. Firstly, convenience sampling was used to access participants, and although this allowed a large number of participants to be recruited, this approach may have resulted in sampling bias. Secondly, because participation was voluntary, the study was open to participation bias, with those with more knowledge, or positive and negative views on the subject potentially more likely to participate. Thirdly, participants of the survey are not representative of the whole UK population, with the majority Caucasian, university educated and heterosexual. Fourthly, the findings of the study are survey based which carries limitations. Closed questions were predominantly used to allow the generation of quantitative data and statistical analysis. However, this approach prevented opinion justification and qualitative analysis, limiting the depth of conclusions drawn. For example, the significant majority of participants was in favour of and would consider ED; however, we know this does not translate in the vast majority of them donating.

Future research should concentrate on face-to-face interviews of the general public. This would allow for the interviewer to investigate and gain in-depth qualitative justification for their answers, enabling more detailed exploration into this complex topic. Particular focus should be on recruiting those from different religious and ethnic backgrounds, as well as same-sex couples. Additionally, face-to-face or video interviews would allow for clarification of question meaning prior to answering to reduce missing data and bias from misinterpretation.

## Conclusions

There is currently an overwhelming lack of knowledge of the practices of egg donation and egg sharing among healthcare professionals. This study has perhaps unsurprisingly shown this is mirrored by the general public. Although the reasons for low egg donor numbers in the UK are likely to be multi-factorial, this lack of knowledge could be contributing to the current donor shortfall in the UK, especially given the high approval rating for the programme.

With studies confirming significant support towards egg donation and egg sharing from both healthcare professionals and the general public in the UK, an emphasis should be placed on campaigns to inform women of this option, for both fertility treatment and social egg freezing, while eliminating any misconceptions, based on theoretical concern instead of scientific fact. This approach could lead to egg share numbers increasing again in the UK, thus benefitting these patients as well as the egg donor shortage.

## Supplementary data

Supplementary data are available at *Human Reproduction* online.

## Data availability

The data underlying this article will be shared on reasonable request to the corresponding author.

## Authors’ roles

T.B.-M., M.Y.-T., N.S. and M.J. were responsible for the study design. T.B.-M. and J.H. were responsible for collecting the data using questionnaires and inputting the data onto the online tool. T.B.-M., J.H. and M.Y.-T. were responsible for data analysis. T.B.-M. and J.H. were responsible for manuscript design, drafting and revision. B.J., S.S., P.A., K.M., J.N.-T. and D.N. were responsible for providing important intellectual input into the work and preparation, drafting and final approval of the manuscript during initial submissions and all revisions. M.Y.-T. is a fertility specialist and the guarantor of this paper and accepts full responsibility for the work and conduct of the study.

## Funding

No external funds were used for this study.

## Conflict of Interest

The authors have no conflicts of interest.

## Supplementary Material

deab157_Supplementary_Data
