# Supplementary material for: Exploring the knowledge and attitudes of women of reproductive age from the general public towards egg donation and egg sharing: a UK-based study
Source: Hum Reprod. 2021 Jul 6;36(8):2189–201. doi: 10.1093/humrep/deab157 (PMC8648294; doi:10.1093/humrep/deab157)
Supplement: deab157_Supplementary_Data [file deab157_supplementary_data.docx]

General Public's View on Egg Donation and Egg Sharing - Updated

Start of Block: Default Question Block

Q40 **A survey of women's attitude and knowledge of egg donation and egg sharing**

 *You have been invited to take part in a research survey investigating women's knowledge and attitudes towards egg donation and egg sharing.* ***Why should I take part in the survey?***
  
 *If you are aged over 18 years, then by filling out this survey you may help other women to have children by being better informed about their fertility choices. The survey will take 5-10 minutes to complete.*
  
 *All responses are anonymous and you will not give any identifiable information.*

Q41 I confirm that I am a **woman**aged **18 years or older** and I **live in the United Kingdom.**

 Please click **yes** to continue the survey.

 Thank you in advance.

- Yes (1)

| Page Break |  |
| --- | --- |

Q1 How old are you?

- 18-25 years old (1)
- 26-30 years old (2)
- 31-35 years old (3)
- 36-40 years old (4)
- 41-45 years old (5)
- >45 years old (6)

Q2 Which best describes your ethnicity?

- White - British (4)
- White - Irish (5)
- White - Other (6)
- Mixed Ethnicity - White/Black Caribbean (7)
- Mixed Ethnicity - White/Black African (8)
- Mixed Ethnicity - White/Asian (9)
- Mixed Ethnicity - Other (please specify) (10) ________________________________________________
- Asian/Asian British - Indian (11)
- Asian/Asian British - Pakistani (12)
- Asian/Asian British - Bangladeshi (13)
- Asian/Asian British - Chinese (14)
- Other Asian Ethnicity (please specify) (15) ________________________________________________
- Black - African (16)
- Black - Caribbean (17)
- Black - British (18)
- Arab (19)
- Other Ethnic Group (please specify) (20) ________________________________________________

Q3 What is your relationship status?

- Single (1)
- In a relationship (unmarried) (2)
- Married (3)

Q4 What best describes your sexual orientation?

- Heterosexual (1)
- Homosexual (2)
- Bisexual (3)
- Other (4)

Q42 Do you have children?

- Yes (1)
- No (2)

Q5 What is your religious background?

- None (1)
- Christian (2)
- Muslim (3)
- Jewish (4)
- Hindu (5)
- Other (please specify) (6) ________________________________________________

Q6 What is your employment status?

- Employed full time (1)
- Employed part time (2)
- Student (3)
- House-wife/husband (4)
- Unemployed (5)

Q7 What occupation category best describes you?

- Architecture and engineering (1)
- Arts, design and media (2)
- Business and finance (3)
- Cleaning and maintenance (4)
- Computing (5)
- Construction, installation and repair (6)
- Education and training (7)
- Farming, fishing and forestry (8)
- Food preparation and related (9)
- Healthcare professional (10)
- House-wife/husband (11)
- Legal (12)
- Management (13)
- Office and administrative support (14)
- Personal care and service (15)
- Sales and related (16)
- Transportation (17)
- Other (please specify) (18) ________________________________________________

Q8 What is your educational level?

- GCSEs (1)
- A-levels (2)
- College diploma/apprenticeship (3)
- University degree (4)
- Postgraduate degree (5)

Q9 What is your annual salary before deductions?

- (1)
- £30,000-£49,999 (2)
- £50,000-£99,999 (3)
- >£100,000 (4)

| Page Break |  |
| --- | --- |

Q10 Please rate each of the following goals 1-5 (1 = not important, 5 = very important) that you would CURRENTLY consider to be important to you

|  | 1 (1) | 2 (2) | 3 (3) | 4 (4) | 5 (5) |
| --- | --- | --- | --- | --- | --- |
| Career (1) |  |  |  |  |  |
| Education (2) |  |  |  |  |  |
| Travel (3) |  |  |  |  |  |
| Meeting a partner (4) |  |  |  |  |  |
| Starting a family (5) |  |  |  |  |  |

Q11 Please rate each of the following goals 1-5 (1 = not important, 5 = very important) that you would consider to be important to you IN THE FUTURE

|  | 1 (1) | 2 (2) | 3 (3) | 4 (4) | 5 (5) |
| --- | --- | --- | --- | --- | --- |
| Career (1) |  |  |  |  |  |
| Education (2) |  |  |  |  |  |
| Travel (3) |  |  |  |  |  |
| Meeting a partner (4) |  |  |  |  |  |
| Starting a family (5) |  |  |  |  |  |

Q12 Have you thought about the decline of fertility with age?

- Yes, frequently (1)
- Yes, occassionally (2)
- Yes, rarely (3)
- Not at all (4)

Q13 At what age do you believe female fertility starts to significantly decline?

- 20-24 years (1)
- 25-29 years (2)
- 30-34 years (3)
- 35-39 years (4)
- 40-44 years (6)
- 45 and older (7)

Q14 At what age would you ideally like to start a family?

- I do not want to have children (1)
- <20 years (2)
- 20-25 years (3)
- 26-30 years (4)
- 31-35 years (5)
- 36-40 years (6)
- 41-45 years (7)
- >45 years (8)

Q15 What would you estimate the UK national average IVF pregnancy rate to be per embryo transfer at the age of 40?

- 0-10% (1)
- 10-20% (2)
- 20-30% (3)
- 30-40% (4)
- 40-50% (5)
- >50% (6)

| Page Break |  |
| --- | --- |

Q16 Egg donation is a process by which a woman donates her eggs to another woman who cannot have a child with her own eggs.
In the United Kingdom (UK) egg donors can be either altruistic donors (a volunteer who donates eggs to an unknown recipient without financial reward) or known donors (donates eggs to a known recipient). Financial payments for egg donation in the UK are illegal.
The process of egg donation involves daily hormone injections to stimulate the ovaries to produce multiple eggs, and then a minor surgical procedure to collect the eggs.


Prior to answering this questionnaire, how much knowledge did you have regarding egg donation programmes?

- No knowledge (1)
- Little knowledge (2)
- Some knowledge (3)
- Significant knowledge (4)

Q17 Do you agree with the principle of egg donation?

- Yes (1)
- No (2)
- Unsure (3)

Q18 Would you consider donating your eggs altruistically as an anonymous donor?

- Yes (1)
- No (2)
- Unsure (3)

Q19 Would you consider donating your eggs to a close friend or relative as a known donor?

- Yes (1)
- No (2)
- Unsure (3)

Q20 Hypothetically, if you were to donate eggs, what would be your main motivation?

- Altruism (1)
- Financial (2)
- Family/friend having fertility problems (3)
- Passing on my genetic material (4)
- To develop a relationship with an infertile couple (5)
- None of the above (6)

Q22 Hypothetically, if you were to donate your eggs, what would be your main concern?

- The medical procedures I would need to go through (1)
- Potential future contact with the child (2)
- The woman receiving my eggs would be too old (3)
- The woman receiving my eggs might be in a same-sex relationship (4)
- The egg donation not working (5)
- Taking time off work (6)
- Religious reasions (7)
- None of the above (8)

Q23 In the UK in 2005, legislative changes meant a child born as a result of egg donation could find out the identity of the egg donor when they reach 18 years of age - do you agree with this?

- Yes (1)
- No (2)
- Unsure (3)

Q21 Would this legislative change stop you from donating your eggs?

- Yes (1)
- No (2)
- Unsure (3)

Q24 Hypothetically, if you donated your eggs, would you want future contact with the couple you donated to?

- Yes (1)
- No (2)
- Unsure (3)

Q25 Hypothetically, if you donated your eggs, would you want future contact with any children resulting from your donation?

- Yes (1)
- No (2)
- Unsure (3)

Q26 If you were unable to conceive and you were advised your only realistic chance of a child was with an egg donor, would you pursue this option?

- Yes (1)
- No (2)
- Unsure (3)

Q27 There is currently a significant shortage of volunteer egg donors in the UK. The UK caps compensatory payments for egg donation at £750 (travel costs, child care etc.), whilst in countries such as the USA, financial reward for egg donation can be as high as $10,000 - do you agree with the compensatory limit of £750 in the UK?

- Yes (1)
- No (2)
- Unsure (3)

Q44 If the compensatory limit were to be increased, would you be more motivated to donate eggs?

- Yes (1)
- No (2)
- Unsure (3)

Q28 Currently to avoid long waiting lists for donor eggs, a significant proportion of couples from the UK are seeking fertility treatment with donor eggs abroad. Do you consider this to be a significant issue?

- Yes (1)
- No (2)
- Unsure (3)

Q29 Should patients requiring donor eggs as part of their IVF treatment have this available to them on the NHS?

- Yes (1)
- No (2)
- Unsure (3)

| Page Break |  |
| --- | --- |

Q45
The UK egg sharing programme is a scheme whereby a fertility patient undergoing IVF for her own needs gives half her eggs to an anonymously matched recipient in exchange for free fertility treatment

Q30 Prior to answering this questionnaire how much did you know of the egg sharing programme?

- No knowledge (1)
- Little knowledge (2)
- Some knowledge (3)
- Significant knowledge (4)

Q31 Do you believe egg sharing is a useful, welcome addition to the field of fertility?

- Yes (1)
- No (2)
- Unsure (3)

Q32 Do you believe egg sharing is a viable solution to solving the worldwide shortage of donor eggs?

- Yes (1)
- No (2)
- Unsure (3)

Q33 Do you see an ethical difference between egg share donors receiving free fertility treatment and a commercial donor being paid by fertility clinics to donate their eggs?

- Yes (1)
- No (2)
- Unsure (3)

Q34 In your opinion, should egg sharing take place?

- Yes (1)
- No (2)
- Unsure (3)

Display This Question:

If In your opinion, should egg sharing take place? = No

Q46 Why not?

________________________________________________________________

Q48 LAST PAGE! :)

Q35 Hypothetically, if you needed IVF to have a child would you consider egg sharing?

- Yes (1)
- No (2)
- Unsure (3)

Q36 Hypothetically, if you were a single female in the UK and wanted to have children in the future, you could freeze your eggs for free for potential future use if you donated half to an anonymous recipient. Were you aware of this option?

- Yes (1)
- No (2)

Q37 In reference to the previous question, do you think this is a good option?

- Yes (1)
- No (2)
- Unsure (3)

Q38 Please GRADE the following potential benefits of egg sharing according to how significant they are from 1-5, where 1 = insignificant and 5 = very significant (benefits can be given the same grade)

|  | 1 (1) | 2 (2) | 3 (3) | 4 (4) | 5 (5) |
| --- | --- | --- | --- | --- | --- |
| Unlike with volunteer egg donors, with egg sharing no third party needs to go through invasive procedures, such as egg collection, since the donor needs to undergo these procedures anyway for her own fertility treatment (1) |  |  |  |  |  |
| Those without access to NHS (government) funded IVF who cannot afford to pay for their treatment get fertility treatment they would otherwise have not been able to access (2) |  |  |  |  |  |
| It allows a person suffering with infertility to help a person in a similar situation (3) |  |  |  |  |  |
| It provides a realistic solution to an acute shortage of eggs (4) |  |  |  |  |  |
| Currently, due to long waiting lists for donor eggs in the UK women are seeking fertility treatment abroad in often unregulated clinics, egg sharing could reduce women seeking treatment abroad (5) |  |  |  |  |  |

Q39 Please GRADE the following potential issues of egg sharing according to how significant they are from 1-5, where 1 = insignificant and 5 = very significant (benefits can be given the same grade)

|  | 1 (1) | 2 (2) | 3 (3) | 4 (4) | 5 (5) |
| --- | --- | --- | --- | --- | --- |
| The donor is only agreeing to share her eggs to gain access to fertility treatment (1) |  |  |  |  |  |
| Concern for the psychological well-being of egg share donors whose own treatment is unsuccessful (2) |  |  |  |  |  |
| The egg share donor might need to undergo repeated IVF cycles and egg collections to collect enough eggs for her and the recipient (3) |  |  |  |  |  |
| Concern that egg sharing could reduce the chances of the donor conceiving as she is donating half her eggs (4) |  |  |  |  |  |
| The recipient of the donor eggs having to pay for treatment when the donor receives treatment for free (5) |  |  |  |  |  |

End of Block: Default Question Block
